# Silicone vs. Silicon/Silica in Intraoral Healing: A Systematic Review

**DOI:** 10.3390/ma19071425

**Published:** 2026-04-02

**Authors:** David Parker, Aditi Bopardikar, Georgios E. Romanos

**Affiliations:** Department of Periodontics and Endodontics, School of Dental Medicine, Stony Brook University, Stony Brook, NY 11794, USA; aditi.bopardikar@stonybrook.edu

**Keywords:** silicone, silicon, silica, bioactive glass, intraoral healing

## Abstract

In the oral environment, silicone (polysiloxane) supports healing by creating low-permeability interfaces that limit microleakage, whereas silicon/silica systems support healing via hydroxyapatite nucleation. We synthesized human evidence on intraoral healing associated with silicone and silicon/silica-based materials and assessed translational differences between preclinical models and clinical settings. A systematic review (1990-September 2025) identified 14 clinical studies of bioactive glass (BAG) that met the inclusion criteria. Periodontal outcomes included probing depth (PD), clinical attachment level (CAL), and radiographic fill; endodontic outcomes included the periapical index (PAI). Human BAG studies showed periodontal benefits versus controls in intrabony defects, with reduced PD, improved CAL, and greater radiographic fill. For endodontic healing, a multicenter randomized clinical trial reported improved PAI at 12 months in both the zinc-oxide-eugenol and silicone-sealer groups without a significant between-group difference. The literature supports a functional split: silicone primarily provides sealing and permissive healing, whereas silicon/silica-based materials support signaling, interfacial bonding, and regenerative healing. Clinically, BAG appears most relevant for contained periodontal intrabony defects, whereas silicone sealers should be viewed primarily as stable sealing adjuncts to well-executed root canal therapy.

## 1. Introduction

The quality of the interface formed between biomaterials, saliva, and oral tissues determines intraoral healing, defined here as the resolution of inflammation and the establishment of a stable hard- and/or soft-tissue interface within the oral cavity after treatment. In this review, silicone refers to polysiloxane-based polymers used principally in endodontic sealers; silicon refers to the elemental basis of silicon-containing biomaterials; silica refers to silicon dioxide (SiO_2_) networks, including glass-derived or mesoporous silica phases; and bioactive glass refers to silicon-containing glass formulations that dissolve under physiological conditions and form a hydroxycarbonate apatite (HCA) or related apatite layer at the tissue interface. This intraoral context matters because saliva, biofilm, and masticatory loading can alter how material–tissue interfaces form and persist in ways that are not fully captured by simplified laboratory models. Regarding endodontics, reducing the microbial and inflammatory burden at the periapical areas and permitting host-driven resolution of apical periodontitis are accomplished through silicone-based root canal sealers designed to limit microleakage by forming a stable, low-permeability barrier along the canal wall. In periodontics, the ability of silicon-containing bioactive glasses and related silicon/silica constructs is valued because they dissolve under physiological conditions, promote nucleation of an HCA or bone-like apatite layer that bonds directly to bone and, to a lesser extent, soft tissues, and release bioactive ions. The two classes—silicone vs. silicon/silica materials—therefore represent distinct therapeutic strategies: sealing and permissive healing versus signaling and regenerative bonding.

Translational and clinical evidence for bioactive glass (BAG) in periodontal intrabony and furcation defects has accumulated over the course of nearly three decades. This includes randomized controlled trials comparing BAG to open flap debridement (OFD), demineralized freeze-dried bone allograft (DFDBA), autogenous bone, and adjunctive biologics [1,2,3]. Meta-analysis and systematic reviews suggest that while the effect sizes may be modest at the defect level and attenuated by heterogeneity in defect morphology, flap management, and follow-up, BAG improves probing depth (PD) reduction and clinical attachment level (CAL) gain relative to OFD alone [4]. Mechanistic studies, at the same time, show that BAG and related silicon-rich glasses release ortho-silicic acid and calcium/phosphate ions that drive rapid HCA formation in simulated body fluid (SBF) and stimulate osteogenic markers in osteoblasts and periodontal ligament (PDL) fibroblasts, thus supporting a biological rationale for interfacial bonding [5,6,7]. These periodontal findings have also been synthesized in more recent reviews of BAG-based regeneration [8,9].

The intraoral evidence base, by comparison, for silicone-based root canal sealers focuses less on regenerative potential and more on postoperative pain, sealing ability, and periapical healing. Similar improvements in periapical index (PAI) scores at 12 months reinforce the view that, in well-performed root canal treatment, sealer chemistry may be less critical than the overall quality of chemo-mechanical preparation and obturation, as shown in a multicenter randomized controlled trial (RCT) comparing silicone and zinc oxide–eugenol (ZOE) sealers [10]. Network meta-analyses and clinical trials comparing calcium silicate, epoxy resin, ZOE, and bioceramic sealers similarly report no clinically meaningful differences in success rates or postoperative pain [11,12,13,14,15]. Nonetheless, the bioactive, degradable behavior of silicon/silica constructs contrasts sharply with silicone sealers, which are important exemplars of a “non-degradable sealing” approach.

Several translational questions remain unresolved. First, much mechanistic work is conducted in SBF and/or static culture conditions that do not replicate the ionic composition, protein content, and flow conditions of saliva [5,6,7,16,17,18,19]. Second, questions are raised regarding how well the observed regenerative benefits translate to human intraoral environments, as animal models (often rat or murine periodontal defects) differ substantially from human intrabony defects in size, geometry, masticatory loading, and microbial ecology [16,18,20,21,22]. Third, the direct contribution of silicone sealer chemistry to cementum or ligament regeneration is rarely measured, and endodontic outcomes are generally evaluated in periapical bone, not in periodontal ligament or soft tissues [10,11,12,13,14,15].

Although these applications are clinically distinct, both depend on how a biomaterial–tissue interface supports intraoral healing. The present systematic review was therefore designed to synthesize human clinical evidence on intraoral healing associated with silicone and silicon/silica-based materials, focusing on periodontal regeneration around intrabony defects and periapical healing following endodontic treatment. We aimed to link, within this framework, clinical outcomes (PD, CAL, radiographic fill, PAI) to mechanistic evidence of apatite formation, ion release, and interfacial bonding, and to clarify how differences between animal and human models, and between SBF and saliva, may constrain the translation of promising preclinical findings. Prior BAG systematic reviews and broader narrative analyses [4,8,9] were built on as we sought to articulate a novel conceptual “split” between silicone as a sealing material and silicon/silica as signaling and bonding materials, and to identify specific experimental and clinical strategies to bridge the gap between preclinical regeneration and durable intraoral performance. The key novelty of this review is the integration of these clinically separate material classes within a single intraoral healing framework that distinguishes barrier-mediated healing from dissolution-mediated signaling and bonding. This framing is clinically relevant because it helps explain when a clinician should prioritize stable sealing and infection control, and when a bioactive, defect-filling material may offer added regenerative value.

## 2. Materials and Methods

This review protocol was registered in PROSPERO on 3 December 2025 (CRD420251164687): https://www.crd.york.ac.uk/PROSPERO/view/CRD420251164687 (accessed on 28 February 2026).

The protocol is publicly accessible through the PROSPERO record above. No substantive post-registration amendments changed the review question or primary outcomes; the operational use of a curated corpus, as described below, reflects how the planned search was executed rather than a change in the review scope.

This systematic review followed the principles of PROSPERO registration and PRISMA guidance for framing the research question, pre-specifying eligibility criteria, and reporting search and selection processes. For clarity, the review was organized a priori into two indication-specific clinical streams within one protocol: periodontal healing with silicon/silica-based bioactive materials and endodontic periapical healing with silicone-based sealers. The predefined question asked in intraoral sites (periodontal or endodontic) in humans, how do silicone-based sealers and silicon/silica-based bioactive constructs compare in terms of interfacial bond formation and healing outcomes, and how do these outcomes relate to mechanistic data from in vitro and animal models? This review was conducted and reported in accordance with PRISMA 2020, and the completed PRISMA 2020 checklist accompanies the submission as Supplementary Material.

Studies were deemed eligible if they evaluated silicone-based endodontic sealers or silicon/silica-based materials, including particulate BAG, BAG putty, or glass-containing composites, in the context of healing in the oral cavity. Randomized controlled trials (RCTs), controlled clinical trials, prospective cohort studies, and relevant mechanistic studies linked to intraoral applications (e.g., BAG tested in PDL fibroblasts or dentin/cementum interfaces) were considered. For periodontal indications, primary outcomes were PD reduction, CAL gain, and radiographic defect fill in intrabony or furcation defects [1,2,3,23,24,25,26,27,28,29]. For endodontic indications, primary outcomes were periapical healing quantified by the PAI, success/failure rates, and, when available, time-dependent changes in lesion size [10,11,12,13,14,15]. Mechanistic endpoints included HCA or apatite layer formation in SBF or modified media, ion-release kinetics for Si, Ca, P, Mg, or Li, osteogenic marker expression, and evidence of interfacial bonding between glass-derived layers and dentin, cementum, or bone [5,6,7,16,17,18,19,20,21,22].

Two reviewers (D.P. and A.B.) independently screened titles and abstracts, assessed full texts for eligibility, and resolved disagreements by discussion, with adjudication by G.E.R. when required. No automation tools were used during screening or selection. The protocol-defined electronic sources were MEDLINE, Embase, and the Cochrane Library for the period 1990 to September 2025; however, the search was operationally executed through a curated corpus consisting of the Abushahba et al. BAG clinical corpus [8], the studies represented in the present reference set, and backward reference-list screening of eligible articles. Search terms were mapped to MEDLINE, Embase, and Cochrane syntax with the harmonized Boolean strategy (“bioactive glass” OR “Bioglass 45S5” OR “calcium sodium phosphosilicate” OR silicon OR silica) AND (“periodontal defect” OR intrabony OR furcation OR “apical periodontitis” OR “root canal sealer” OR “silicone sealer”). No database-specific filters, language restrictions, or study-design search limits were applied beyond the 1990 to September 2025 window and the oral/intraoral relevance criteria. Duplicate citations were removed manually before screening. At the title/abstract screening, records were excluded for non-oral indications, non-human or preclinical-only design, lack of a silicone- or silicon/silica-based intervention of interest, review/commentary format, or absence of relevant healing outcomes. Full-text assessment was then performed on all potentially eligible reports. Information sources comprised the curated Abushahba et al. BAG clinical corpus, the studies represented in the present reference set, and backward reference-list screening of eligible articles; no additional trial registries, organizational websites, or gray literature repositories were searched. The final search update and citation cross-check were completed in September 2025. Because the review relied on a curated corpus rather than direct live database execution, separate database-specific search strings and database-level hit counts were not retained.

Data extracted from periodontal trials included defect characteristics (intrabony vs. furcation), material type and formulation (particulate BAG, BAG putty, BAG + DFDBA, BAG + enamel matrix derivative, BAG vs. membrane), sample size, follow-up duration, and clinical and radiographic outcomes (PD, CAL, defect fill, furcation closure) [1,2,3,23,24,25,27,28,29,30]. We recorded sealer type (ZOE, silicone, epoxy resin, calcium silicate), obturation technique, lesion baseline PAI or lesion size, follow-up, and healing or success criteria from endodontic studies. Postoperative pain and analgesic consumption were also abstracted, where reported [10,11,12,13,14,15,31]. For composition (e.g., 45S5, Li-modified or Mg-modified silica, mesoporous silicon), test solutions (SBF vs. artificial saliva), temporal profile of HCA formation, ion-release profiles, and cellular or tissue responses, mechanistic studies were abstracted [5,6,7,16,17,18,19,20,21,22]. Data extraction was performed independently by D.P. and A.B. with a predefined extraction framework that captured study design, population or defect characteristics, intervention and comparator details, follow-up, and reported clinical or mechanistic outcomes. Discrepancies were reconciled by consensus review of the source report. Study investigators were not contacted because the required outcomes were available in the published articles, and no automation tools were used. Funding-source/conflict disclosures and any reported precision measures were also extracted when available; when these fields or other methodological details were unclear or absent in the source reports, they were recorded as not reported and no values were imputed.

Using the RoB 2 framework, the risk of bias in periodontal RCTs was considered with attention to randomization, allocation concealment, blinding of outcome assessors, completeness of follow-up, and selective reporting [4]. For the endodontic randomized sealer trial, the same core RoB 2 domains were considered together with the consistency of outcome definitions and time points [10]. Mechanistic studies were appraised qualitatively, focusing on the ecological validity of test media (SBF vs. saliva), flow conditions, and whether specimen geometry approximated clinical defects. A quantitative meta-analysis was not repeated, given the limited number of human RCTs per comparison and substantial heterogeneity in defect characteristics; instead, we synthesized findings narratively, emphasizing the direction and magnitude of effects and how they align or conflict with mechanistic expectations. Risk-of-bias assessment was performed independently by D.P. and A.B., with disagreements resolved by consensus and adjudication by G.E.R. when needed; no automation tools were used for this step. A structured study-level summary of these judgments is provided in the Results.

Effect measures were recorded exactly as reported by the source studies and were summarized as study-level mean changes, between-group differences, percentage defect fill, PAI changes, postoperative pain frequency or intensity, or success and failure rates, as applicable. Studies were grouped for synthesis by indication (periodontal vs. endodontic), material class, comparator, and follow-up interval. No numeric data conversions, imputations, or de novo pooled meta-analysis were undertaken; when reporting was incomplete, findings were retained narratively. Results are presented in the narrative text, structured tables, and the PRISMA flow diagram. Heterogeneity, possible reporting bias, and certainty were evaluated qualitatively by comparing defect morphology, adjunctive therapy, material formulation, follow-up duration, indirectness, and consistency across reports; no subgroup meta-analysis, meta-regression, or quantitative sensitivity analysis was performed.

## 3. Results

### 3.1. Periodontal Intrabony and Furcation Defects Treated with Bioactive Glass

**Figure 1:**
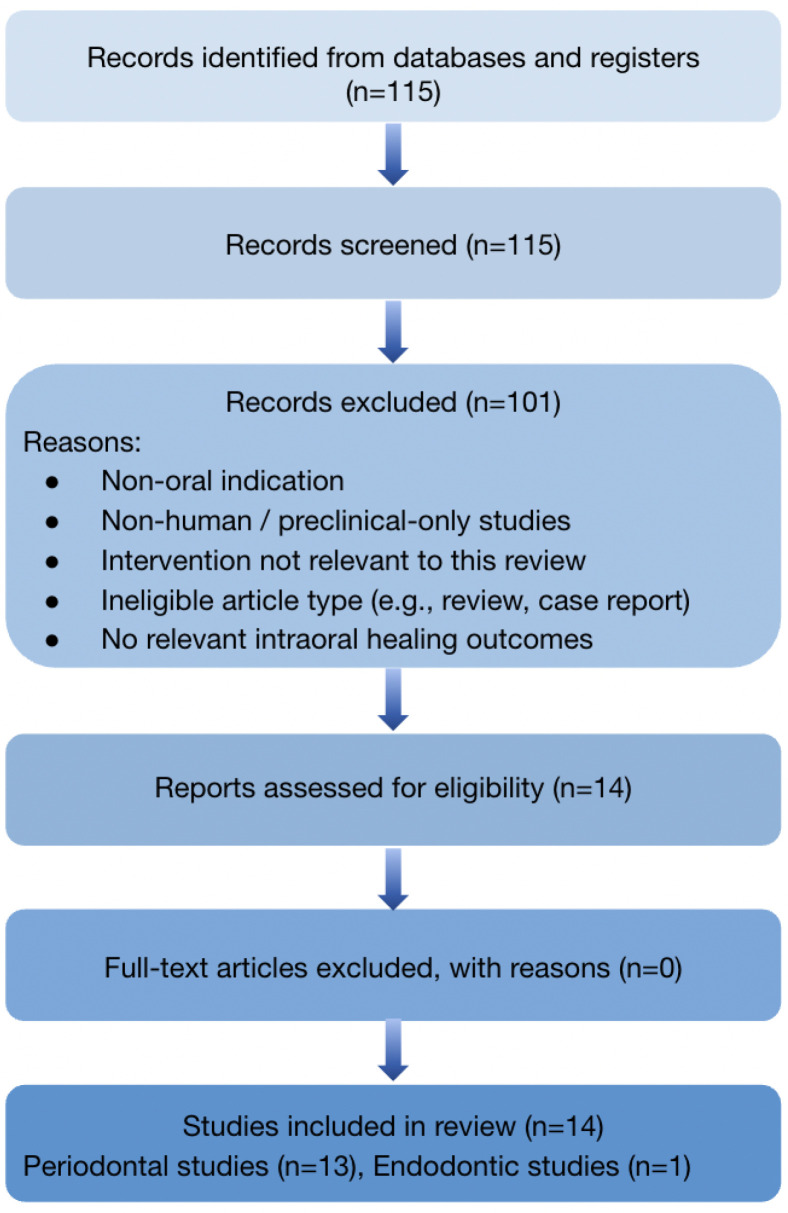
PRISMA 2020 flow diagram detailing study identification and selection for this review. The figure summarizes the number of records screened, the full-text articles assessed for eligibility, the final included studies, and the principal exclusion categories at the title/abstract review, including non-oral indication, non-human/preclinical-only design, non-silicone- or non-silicon/silica-based biomaterial focus, review/commentary format, and the absence of relevant healing outcomes.

The search and selection process is summarized in Figure 1: 115 records were screened, 14 full-text reports were assessed for eligibility, and all 14 were included in the review. Records excluded during title/abstract screening were removed because they addressed non-oral indications, were non-human or preclinical-only studies, did not evaluate a silicone- or silicon/silica-based intervention relevant to the review question, were review/commentary articles, or lacked relevant healing outcomes. No otherwise eligible full-text reports were excluded after full-text assessment.

**Figure 2:**
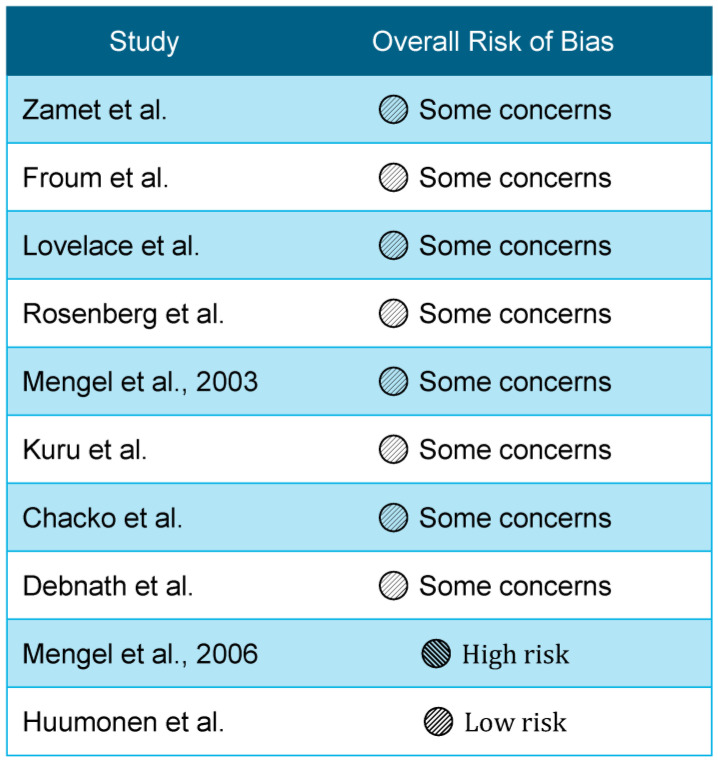
Most periodontal trials were judged as having some concerns due to small sample sizes and limited reporting. The studies shown are Zamet et al. [1], Froum et al. [2], Lovelace et al. [3], Rosenberg et al. [30], Mengel et al., 2003 [25], Kuru et al. [23], Chacko et al. [28], Debnath et al. [29], Mengel et al., 2006 [24], and Huumonen et al. [10]. Mengel et al., 2006 [24] was assessed as having a high risk of bias due to attrition and concerns regarding long-term comparability. Huumonen et al. [10] was assessed as having a low risk of bias overall.

Particulate bioglass (PerioGlas^®^ (NovaBone Products, LLC., Alachua, FL, USA)) used as a grafting material yields greater radiographic defect fill and trends toward improved PD reduction and CAL gain compared with OFD alone, as demonstrated by early human RCTs of BAG in periodontal intrabony defects. Zamet et al. reported that in 20 patients with 44 intrabony defects, BAG-treated sites showed significantly greater increases in radiographic density and defect volume fill by computer-assisted densitometric image analysis (CADIA), with clinically relevant improvements in PD and probing attachment level (PAL) compared with surgically debrided controls [1]. Froum et al. similarly observed that BAG-synthetic bone graft particles achieved greater defect fill than OFD in 59 defects, with both groups exhibiting improved PD and CAL but with a trend favoring BAG for hard-tissue regeneration [2]. The study-level risk-of-bias judgments for the included periodontal and endodontic trials are summarized in Figure 2.

Subsequent RCTs explored BAG in comparison with DFDBA, autogenous bone, and membrane-based guided tissue regeneration (GTR), as well as in combination with enamel matrix derivative (EMD). Lovelace et al. compared BAG with DFDBA in paired intrabony defects, reporting comparable bone fill (approximately 62%) and defect resolution in both groups, indicating that BAG performs at least as well as a widely accepted allograft in these defects [3]. Rosenberg and co-researchers evaluated BAG granules of uniform size in interproximal intrabony defects and found favorable gains in PD, CAL, and radiographic defect fill, confirming the reproducibility of earlier findings [30]. Kuru et al. investigated wide intrabony periodontal defects treated with EMD alone or EMD plus BAG; both approaches improved PD, CAL, and radiographic bone levels over eight months, with a numerical but not clearly clinically decisive advantage in attachment gain and defect fill for the combination group [23]. Mengel and colleagues conducted a five-year clinical and radiological study comparing a bioabsorbable membrane and BAG in intrabony defects in generalized aggressive periodontitis, finding that both modalities achieved substantial and durable improvements, with BAG representing a viable long-term alternative [24,25].

Across the included periodontal trials, BAG was generally associated with improved PD, CAL, and radiographic defect fill relative to OFD, while comparisons with DFDBA, autogenous bone, membranes, or EMD were either comparable or numerically favorable to BAG depending on defect configuration and follow-up.

Data on furcation defects were limited but suggested favorable outcomes in Class II furcation lesions, including gains in horizontal and vertical defect dimensions and, in some reports, partial furcation closure.

### 3.2. Silicone-Based Sealers and Periapical Healing

The principal randomized clinical evidence directly addressing silicone versus ZOE sealer chemistry in periapical healing comes from the multicenter RCT by Huumonen and colleagues, in which 199 teeth with apical periodontitis received either a ZOE-based sealer or a silicone-based sealer [10]. At 12 months, mean PAI scores improved from approximately 3.4 to 2.2 in both groups, with no significant difference in healing between ZOE and silicone.

**Figure 3:**
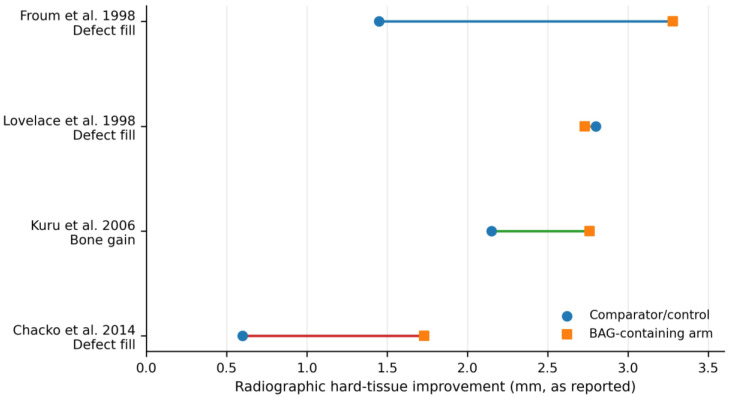
Representative clinical hard-tissue outcomes from selected human periodontal trials. Values are plotted as reported in the original studies for BAG-containing arms and their comparators/controls and correspond to radiographic defect fill (Froum et al. [2], Lovelace et al. [3], Chacko et al. [28]) or radiographic bone gain (Kuru et al. [23]). The colored connecting lines are used only to visually distinguish the individual studies and do not indicate additional categories. The endodontic randomized trial was not plotted because it reported the periapical index (PAI) rather than a directly comparable defect fill or bone gain metric.

Figure 3 summarizes selected human periodontal trials that reported representative radiographic hard-tissue outcomes (defect fill or bone gain, mm) [2,3,23,28]. The visual pattern is consistent with the narrative synthesis, showing generally greater hard-tissue improvement in BAG-containing arms than in control/comparator groups, with Lovelace et al. showing near-equivalent performance versus DFDBA [2,3,23,28].

Synthesis of the included evidence was driven primarily by small periodontal clinical trials involving intrabony or furcation defects and follow-up ranging from 6 months to 5 years, plus one multicenter endodontic randomized trial; accordingly, the contributing studies were clinically heterogeneous despite directional consistency in the main outcomes. Across the periodontal trials, defect morphology (contained intrabony defects versus furcation lesions), treatment protocol (OFD alone, BAG alone, BAG combined with EMD or membranes, and comparisons with DFDBA or autogenous bone), follow-up duration, and selected outcome measurements (PD, CAL, radiographic defect fill, and furcation closure) varied substantially and likely influenced the apparent magnitude of benefit. The endodontic trial used the radiographic PAI as the principal healing outcome at 3 and 12 months, which limits direct comparison with periodontal regenerative endpoints. Across studies, the risk of bias was most often characterized by some concerns because of small sample sizes, limited masking, and variable reporting of allocation concealment. A structured RoB 2 summary is as follows: Zamet et al. [1], Froum et al. [2], Lovelace et al. [3], Rosenberg et al. [30], Kuru et al. [23], Chacko et al. [28], and Debnath et al. [29] were judged as having some concerns; Mengel et al. (2003) [25] was also judged as having some concerns, whereas the 5-year follow-up report (Mengel et al., 2006) [24] was considered at a higher risk because of attrition and long-term comparability concerns; Huumonen et al. [10] was judged as low risk overall, although some performance and follow-up limitations remained. Individual study outcome data are presented in Table 1 exactly as reported; where available, summary statistics and precision measures are shown as mean ± SD, whereas confidence intervals were generally not reported in the older primary trials. A summary of the secondary evidence referenced is provided in Table 2.

No formal quantitative statistical synthesis, subgroup analysis, or sensitivity analysis was performed in this review; accordingly, no pooled summary estimate, confidence interval, or statistical heterogeneity metric was generated. Qualitative comparison suggested that heterogeneity was driven mainly by defect morphology, adjunctive therapy, material formulation, and follow-up duration. Because the evidence base was curated and no pooled model was fitted, reporting bias was considered only qualitatively. Overall certainty was judged as moderate-to-high for the conclusion that BAG provides modest periodontal benefit, moderate for the direct translation of mechanistic silicon or silica findings to human intraoral performance, and moderate-to-high for the conclusion that currently available non-bioactive sealer chemistries produce broadly similar short-term periapical healing when treatment quality is high.

## 4. Discussion

Confined to a curated but clinically relevant set of human and preclinical studies, this systematic review supports a functional distinction between silicone-based sealers and degradable silicon/silica-based constructs in intraoral healing. Silicone sealers are engineered to be dimensionally stable, hydrophobic, and minimally soluble, with the primary aim of preventing reinfection and providing a durable barrier; this exemplifies a “sealing and permissive healing” strategy. Rather than active regenerative signaling arising from the sealer itself, healing of apical periodontitis depends largely on the efficacy of microbial control during chemo-mechanical preparation. Provided a competent seal is achieved, the multicenter RCT showing equivalent PAI improvements for ZOE and silicone sealers reinforces that the specific chemistry of a non-bioactive sealer exerts only modest influence on periapical healing outcomes [10]. Equivalence should be interpreted as no detectable difference under these study conditions, because the clinical endodontic endpoint here is the radiographic PAI at 12 months, not as proof that sealer chemistry is universally irrelevant, especially over longer follow-up, in cases with persistent intraradicular infection, or in complex anatomy. Mechanistically, this side of the split is consistent with polymer-network stability and low solubility favoring the containment of irritants rather than ion-mediated tissue induction.

Silicon/silica-based BAG and related materials, in contrast, operate via dissolution-driven bioactivity. Their clinical benefits in periodontal intrabony defects—modest but consistent improvements in PD, CAL, and radiographic fill relative to OFD, and performance comparable to DFDBA and autogenous bone—are congruent with mechanistic evidence for surface hydrolysis, silanol formation, rapid HCA-layer formation, and osteogenic ion signaling [1,2,3,4,5,6,7,16,17,18,32]. The ability of silicon-containing glasses and mesoporous silica constructs to modulate osteogenic markers in periodontal and bone-related cells, and to support bone bonding and defect fill in preclinical and clinical models, further underscores their regenerative potential [6,20,21,22]. These materials illustrate a “signaling + interfacial bonding” model, from a conceptual standpoint, in which biomaterial degradation products actively shape the healing microenvironment rather than remaining inert. Without histologic confirmation, apparent fill can reflect repair, long junctional epithelium, or graft remnants rather than new cementum/PDL insertion; the summarized clinical outcomes (PD/CAL/radiographic fill) therefore remain intermediate clinical measures of true periodontal regeneration. This distinction matters clinically because small average gains can look encouraging at the defect level yet translate into limited incremental tooth-level benefit once baseline prognosis, patient plaque control, and maintenance are accounted for. This interpretation is consistent with contemporary reviews describing modest average PD/CAL gains together with appreciable heterogeneity across defect morphology, treatment protocol, and follow-up [4,8,9]. Furcation outcomes appear more variable than contained intrabony defects, where space maintenance and material retention are typically more predictable [2,33].

This review, at the same time, highlights important translational gaps and limitations. Heterogeneity among periodontal RCTs in defect morphology, flap design, adjunctive therapies, and follow-up makes it difficult to isolate the specific contribution of BAG, and meta-analytic effect sizes suggest that while BAG improves PD and CAL compared with OFD, the magnitude of benefit may be limited at the patient level [4,8,9]. Most mechanistic studies use SBF or static tissue-culture conditions that do not adequately model salivary composition, protein adsorption, or dynamic flow. Differences in saliva flow, buffering capacity, enzymatic content, microbial biofilms, occlusal loading, and defect geometry between species and between laboratory models and human sites could alter dissolution kinetics, HCA layer integrity, and the durability of interfacial bonding in humans [5,6,7,16,17,18,19,22]. Many periodontal trials are small and difficult to blind, so modest effect sizes are vulnerable to performance and detection bias, defect-selection effects, and regression to the mean; this is a second-order limitation of internal validity. Saliva is not merely another fluid in this context: the acquired pellicle, biofilm-derived acids, and salivary proteins may destabilize or suppress apatite nucleation that appears robust in plasma-mimicking SBF, so time-to-apatite in SBF may overpredict durable intraoral bonding.

From a practical treatment standpoint, the periodontal evidence suggests that BAG is most reasonably viewed as an adjunct for well-debrided, contained intrabony defects rather than a universal substitute for defect-specific case selection, flap stability, and supportive periodontal care. Outcomes in furcation defects or less-contained morphologies are more variable, partly because material retention and regenerative space maintenance are less predictable. In endodontics, the present data suggest that meticulous disinfection, working-length control, canal preparation, and obturation quality are likely to exert more influence on periapical healing than choosing among currently available non-bioactive sealer chemistries. Taken together, these findings guide clinical practice by favoring BAG-type materials when regenerative defect support is desired in appropriately contained periodontal defects, while reinforcing that silicone sealer selection should not supersede disinfection and sealing quality in root canal therapy.

Dissolution and ion release can be tuned (payload, dose, and rate) to better fit intraoral constraints, as suggested by lithiated porous-silicon nanowires and engineered mesoporous silica platforms. Immunomodulatory effects have also been demonstrated by these systems, including skewing macrophage polarization toward pro-healing phenotypes, which are increasingly recognized as central to successful regeneration. The absence of human data and the reliance on small-animal models with simplified loading and microbial environments, however, mean that their translational value remains speculative. Nanoparticle clearance or persistence, delivery format (granule/coating/putty), local concentration thresholds, and regulatory toxicity requirements may become dominant barriers well before efficacy is tested in humans; thus, platform sophistication is not automatically equivalent to clinical readiness. Therefore, future translation should justify incremental benefit over simpler BAG grafting by demonstrating durability under biofilm challenge and cyclic loading and by predefining clinically meaningful endpoints (tooth survival, need for re-surgery, patient-reported outcomes), rather than short-term osteogenic signaling alone. Related mechanistic literature on mesoporous silica and lithiated porous silicon further supports this signaling framework, but comparative human data for these newer platforms are not yet available [7,18,19,20,21,22].

Regarding endodontics, the relative equivalence of different sealer chemistries in terms of postoperative pain and radiographic success, documented in RCTs, cohort studies, and meta-analyses, implies that efforts to enhance periapical healing may be better directed toward improving disinfection, shaping, and three-dimensional obturation than toward incremental changes in sealer formulation, at least among currently available products [11,12,13,14,15,31]. Future silicon-containing sealers may bridge the gap between sealing and signaling models if they are designed to combine sealing with controlled bioactive ion release. Demonstrating added value will likely require longer follow-up and more granular imaging and clinical outcomes than “pain + 12-month radiograph,” because true material-driven advantage is expected to be small relative to operator and case-mix effects. This is also consistent with secondary evidence showing broadly similar postoperative pain and short-term success across currently available sealer classes despite in vitro differences in cytotoxicity and inflammatory signaling [10,11,12,13,14,15,31].

## 5. Conclusions

A conceptual and functional split in intraoral silicone-related biomaterials is supported by the available evidence. Although the periodontal and endodontic applications are clinically distinct, they are complementary in showing that intraoral healing can be promoted either by stable sealing or by dissolution-mediated signaling and bonding. At present, silicone-based endodontic sealers act as inert, low-permeability barriers that permit resolution of apical periodontitis when combined with adequate disinfection and obturation without substantially altering healing pathways. Degradable silicon/silica-based materials, such as BAG and newer mesoporous or ion-modified constructs, promote intraoral healing by releasing ortho-silicic acid and other ions that drive HCA nucleation, support osteogenic and immunomodulatory responses, and foster interfacial bonding in periodontal defects. Although human RCTs indicate that BAG confers modest but consistent clinical benefits in intrabony periodontal defects compared with OFD, equivalence of silicone and ZOE sealers in periapical healing emphasizes the permissive nature of sealing-based approaches. This review provides a novel contribution in framing silicone vs. silicon/silica as “sealing” versus “signaling + bonding” strategies across periodontal and endodontic contexts, and in integrating mechanistic and clinical data to explain why preclinical successes with silicon-based constructs may attenuate in human trials. Clinically, the findings support BAG as a selective regenerative adjunct in appropriate periodontal defects while underscoring that, for endodontic healing, material choice remains secondary to high-quality infection control and obturation.

## Figures and Tables

**Table 1:** Included human clinical studies of bioactive glass in periodontal defects and silicone-based sealers in endodontics.

Study (Reference)	Indication/ Defect	Design/Sample	Intervention vs. Comparator	Follow-Up	Key Results (As Reported)
Zamet et al., 1997 [1]	Periodontal intrabony defects	Randomized controlled clinical trial; 20 patients; 44 sites	PerioGlas^®^ (bioactive glass) + flap surgery vs. flap surgery alone	3 mo, 6 mo, 9 mo, and 1 yr	At 1 y: CADIA increased with PerioGlas^®^ vs. surgery alone (F = 15.67; *p* < 0.001); PPD/PAL improved in both groups (trend favoring PerioGlas^®^)
Froum et al., 1998 [2]	Periodontal osseous defects (intrabony/furcation)	Randomized controlled clinical trial; 16 adults; 59 defects	Bioactive glass particles vs. open flap debridement	6 mo, 9 mo, and 12 months	At 12 mo: bioactive glass vs. OFD—PD reduction 4.26 vs. 3.44 mm; CAL gain 2.96 vs. 1.54 mm; defect depth reduction 4.36 vs. 3.15 mm; defect fill 3.28 vs. 1.45 mm; recession 1.29 vs. 1.87 mm
Lovelace et al., 1998 [3]	Moderate–deep intrabony defects	Randomized controlled clinical trial; 15 patients; 20 defects	Bioactive glass vs. DFDBA	6 months	At 6 mo: bioactive glass vs. DFDBA—PD reduction 3.07 ± 0.80 vs. 2.60 ± 1.40 mm; CAL gain 2.27 ± 0.88 vs. 1.93 ± 1.33 mm; defect fill 2.73 vs. 2.80 mm; bone fill 61.8% vs. 62.5% (NS)
Rosenberg et al., 2000 [30]	Interproximal intrabony defects	Split-mouth comparative clinical trial; 12 patients (12 pairs of lesions)	Bioactive glass + OFD vs. OFD alone	6 months	At 6 mo: both improved; bioactive glass had greater CAL gain (*p* < 0.01) and greater hard tissue fill at re-entry (*p* < 0.001) vs. OFD
Mengel et al., 2003 [25]	Aggressive periodontitis intrabony defects (1–3 wall)	Prospective randomized trial; 12 patients; 30 defects (15/group)	Bioabsorbable membrane vs. bioactive glass granules (PerioGlas^®^)	6 mo and 12 months	At 12 mo: membrane vs. bioactive glass—PD reduction 4.0 ± 2.1 vs. 3.8 ± 1.9 mm; CAL gain 3.4 ± 2.3 vs. 2.8 ± 1.9 mm; GR increase 0.6 ± 1.5 vs. 1.0 ± 1.4 mm
Mengel et al., 2006 [24]	Aggressive periodontitis intrabony defects (1–3 wall)	5-year follow-up of randomized clinical trial; 16 patients; 42 defects (22 membrane; 20 bioactive glass)	Bioabsorbable membrane vs. bioactive glass granules (PerioGlas^®^)	6 mo annually for 5 years	5 y: membrane vs. bioactive glass—PD reduction 3.6 ± 0.8 vs. 3.5 ± 1.4 mm; CAL gain 3.0 ± 2.0 vs. 3.3 ± 2.1 mm; GR increase 0.6 ± 1.4 vs. 0.2 ± 1.7 mm; radiographic defect fill 47.5% ± 38.3 vs. 65.0% ± 50.5
Kuru et al., 2006 [23]	Wide intrabony defects (chronic periodontitis)	Randomized clinical trial; 23 patients with chronic periodontitis	Enamel matrix derivative (EMD) vs. EMD + bioactive glass	8 months	8 mo: EMD vs. EMD + BG—PD reduction 5.03 ± 0.89 vs. 5.73 ± 0.80; CAL gain 4.06 ± 1.06 vs. 5.17 ± 0.85; radiographic bone gain 2.15 ± 0.42 vs. 2.76 ± 0.69 mm; GR 0.97 ± 0.24 vs. 0.56 ± 0.18
Chacko et al., 2014 [28]	Human periodontal osseous defects (two- and three-wall)	Randomized controlled clinical trial; 10 patients; 20 sites (10/group)	OFD + PerioGlas^®^ (bioactive glass) vs. OFD alone	6 weeks, 3 mo, 6 mo, and 9 months	At 9 mo: PerioGlas^®^ + OFD vs. OFD—PPD reduction 4.4 ± 0.34 vs. 3.2 ± 0.10 mm; CAL gain 4.4 ± 0.21 vs. 3.4 ± 0.11 mm (NS); defect resolution 46.5% vs. 15.3%; defect fill 1.73 mm vs. 0.60 mm
Debnath et al., 2014 [29]	Periodontal infrabony (bony) defects	Randomized controlled study; 18 patients; 30 sites (10/group)	TG1: HA:BG composite + biodegradable membrane; TG2: HAP + membrane; Control: OFD + membrane	6 months	6 mo: defect fill (mm) TG1 2.6 ± 0.66 > TG2 1.6 ± 0.66 > control 0.9 ± 0.7; CAL (baseline→6 mo) TG1 6.3 ± 1.26→3.4 ± 1.11, TG2 4.9 ± 1.64→3.2 ± 1.4, control 3.4 ± 0.66→2.2 ± 0.6
Huumonen et al., 2003 [10]	Apical periodontitis after RCTx	Randomized controlled clinical trial; 199 teeth (multicenter)	Silicone-based sealer (RoekoSeal) vs. zinc oxide–eugenol sealer (Grossman)	3 mo and 12 months	At 12 mo: silicone-based sealer (RoekoSeal) vs. Grossman—PAI improved similarly (Grossman 3.43→2.21; RoekoSeal 3.40→2.26); no significant difference at 3 or 12 mo

Notes: PD = probing depth; CAL = clinical attachment level; PAL = probing attachment level; GR = gingival recession; OFD = open flap debridement; DFDBA = demineralized freeze-dried bone allograft; BG = bioactive glass; HA/HAP = hydroxyapatite; CADIA = computer-assisted densitometric image analysis; PAI = periapical index. Values are reported as mean ± SD unless stated otherwise. Radiographic outcomes reflect defect fill or bone gain as reported by the original authors using study-specific measurement techniques.

**Table 2:** Summary of secondary evidence (systematic reviews/meta-analyses) referenced in this manuscript.

Reference	Type	Scope	Key Conclusions (As Reported)
Motta et al., 2023 [4]	Systematic review (with meta-analysis)	Periodontal intrabony defects; clinical outcomes (PD, CAL) comparing BG to OFD and other grafts/adjuncts	SR/MA: BG vs. OFD shows SMD ~0.5–1 for PD and CAL (statistically significant but clinically small); heterogeneity limits certainty
Cannillo et al., 2022 [9]	Literature review	BGs in periodontal regeneration; in vitro, in vivo, and clinical studies (screened 2006–March 2021)	Literature review (1 January 2006–31 March 2021): many in vitro studies, fewer animal/clinical; overall BGs appear suitable graft materials for periodontal regeneration
Vijayalakshmi et al., 2023 [32]	Short note/commentary	Bioglass composition and proposed mechanism in periodontics	Short note: BG (Na/Ca salts, phosphates, SiO_2_) forms a hydroxycarbonate apatite surface layer in tissue fluids; theorized to promote osteogenesis/bone formation

Notes: BG = bioactive glass; PD = probing depth; CAL = clinical attachment level; OFD = open flap debridement; SMD = standardized mean difference. Key conclusions are summarized as reported by the original authors.

## Data Availability

The data presented in this study are available within this article. Additional extracted data, the study-selection log underlying Figure 1, the predefined extraction framework, and the completed PRISMA 2020 checklist are available from the corresponding author on reasonable request. No analytic code was generated because no new quantitative meta-analysis was performed.

## Supplementary Materials

The following supporting information can be downloaded at: https://www.mdpi.com/article/10.3390/ma19071425/s1. Supporting Information: PRISMA 2020 checklist. Reference [34] is cited in the Supplementary Materials.
